# Repetitive Transcranial Magnetic Stimulation for Intractable Neuropathic Pain Following Post-Traumatic Lumbosacral Plexopathy: A Case Report

**DOI:** 10.3390/bioengineering13030325

**Published:** 2026-03-11

**Authors:** Jae-In You, Jae-Hyung Kim

**Affiliations:** 1 Department of Physical Medicine & Rehabilitation, Eulji University Hospital, Daejeon 35233, Republic of Korea; 20251153@eulji.ac.kr; 2 Department of Physical Medicine & Rehabilitation, Chonnam National University Hwasun Hospital, Jeollanam-do 58128, Republic of Korea; 3 Department of Physical Medicine & Rehabilitation, Chonnam National University Medical School, Gwangju 61469, Republic of Korea

**Keywords:** sacral plexopathy, neuropathic pain, nerve injury, neuromodulation, transcranial magnetic stimulation, motor cortex stimulation

## Abstract

Background: Lumbosacral plexopathy (LSP) is characterized by severe neuropathic pain, motor weakness, and sensory deficits in the lumbosacral plexus region, often leading to significant functional impairment and reduced quality of life. Post-traumatic LSP is particularly challenging to treat due to its neuropathic nature and limited response to conventional pharmacologic therapies. Repetitive transcranial magnetic stimulation (rTMS) is a non-invasive neuromodulation technique that has shown therapeutic potential for chronic neuropathic pain. Case Report: We report the case of a 16-year-old female who developed LSP following multiple pelvic fractures and subsequently exhibited disabling pain, depressive symptoms, and poor quality of life. High-frequency motor cortex rTMS resulted in meaningful clinical improvement in pain intensity (an NRS reduction from 8 to 2), mood, and daily functioning. Conclusions: This suggests the potential role of rTMS as an adjunctive treatment for refractory neuropathic pain secondary to traumatic LSP.

## 1. Introduction

Lumbosacral plexopathy (LSP) manifests as a clinical syndrome marked by severe neuropathic pain, muscle weakness, atrophy, and sensory abnormalities such as paresthesia, hypoesthesia, dysesthesia, and hyperpathia within the lumbosacral plexus’s anatomical bounds [1]. The pain is often refractory and unresponsive to conventional analgesics, presenting both diagnostic and therapeutic challenges. While the lumbosacral plexus is well-protected within the pelvic brim, traumatic LSP is not exceedingly rare and significantly affects patient quality of life (QoL) [1,2]. Post-traumatic LSP represents a particular group of LSP cases often resulting from high-energy trauma (e.g., motor vehicle accidents, falls from height), penetrating injuries (e.g., gunshot or stab wounds), postoperative complications, or compression during childbirth [3]. LSP may also arise secondary to conditions such as retroperitoneal hematomas, aortic dissection or aneurysm, lumbar plexus anesthesia, or iatrogenic ischemia from femoral artery procedures. The incidence of LSP following pelvic trauma is about 0.7%, which increases to 2% in cases with sacral fractures [4]. Patients typically present with low back pain or radiating leg pain, often accompanied by motor weakness, sensory loss, or autonomic symptoms like bowel or bladder dysfunction [2]. Neurological deficits may be isolated or extensive, potentially affecting both the proximal and distal lower extremities, and can be bilateral [1]. Magnetic resonance imaging (MRI) and electrodiagnostic tests are critical for accurate diagnosis, with MRI providing detailed structural information and electrodiagnosis helping to pinpoint lesion location and severity [5].

Neuropathic pain, as defined by the International Association for the Study of Pain, is caused by a lesion or disease affecting the somatosensory system and can result in significant reductions in QoL, as well as functional impairment, depression, and sleep disturbances [6]. Central neuropathic pain is well recognized following events such as a stroke, traumatic brain injury, or spinal cord injury. Peripheral neuropathic pain occurs in conditions including diabetic neuropathy, post-herpetic neuralgia, and LSP. Current treatments encompass pharmacotherapy, nerve blocks, cognitive behavioral therapy, and a variety of physical and psychological interventions, along with acupuncture, massage, and other complementary therapies [7]. However, many patients with neuropathic pain respond inadequately to conventional pharmacologic treatments because of their heterogeneous pathophysiology, highlighting the need for alternative or adjunctive treatment modalities [8]. Common medications such as antidepressants and anticonvulsants are non-selective and commonly lead to central nervous system side effects, including sedation, fatigue, and dizziness [7]. To address these challenges, non-invasive neuromodulation techniques such as transcranial magnetic stimulation (TMS) have gained prominence. TMS modulates cortical excitability and provides indirect pain control. High-frequency repetitive TMS (HF-rTMS) has been reported to be beneficial in various chronic pain conditions, including complex regional pain syndrome [9,10]. Repetitive transcranial magnetic stimulation can either inhibit or excite cortical activity depending on the frequency of stimulation: low-frequency (≤1 Hz) stimulation is generally inhibitory, whereas high-frequency (≥5 Hz) stimulation tends to be excitatory. These actions may modulate thalamo-cortical circuits and affect the affective and cognitive aspects of pain [11].

To date, no prior reports have described the use of rTMS in treating neuropathic pain resulting from traumatic LSP. Previous studies investigating HF-rTMS in chronic neuropathic pain, including complex regional pain syndrome and post-stroke pain, have demonstrated short-term analgesic benefits typically lasting up to one week [12,13,14]. Given the limited therapeutic options available for young patients with severe traumatic neuropathic pain and the potential long-term adverse effects of chronic pharmacologic therapy, identifying safe and effective non-invasive alternatives is of considerable clinical importance. rTMS has demonstrated a favorable safety profile, including in adolescent populations when applied within established safety guidelines, making it a potentially valuable adjunctive therapy in this age group. This study aims to evaluate the clinical efficacy of rTMS in this context, assessing not only pain relief but also potential improvements in depressive symptoms and quality of life, thereby suggesting the potential utility of rTMS as an alternative option for young patients with intractable neuropathic pain.

## 2. Case Report

A 16-year-old female fell from a height of five floors and was admitted to the emergency department with multiple traumatic injuries, including a right sacral fracture, bilateral superior and inferior pubic ramus fractures, right subtrochanteric femur fracture, coccyx fracture, and right calcaneus fracture. Serial external fixation and internal fixation were performed for stabilization. A massive pelvic hematoma was subsequently identified and managed with embolization of the right iliolumbar artery. After stabilization of her general condition, she was transferred to the Department of Rehabilitation Medicine for management of persistent right lower-limb pain and functional rehabilitation.

The patient reported two distinct types of pain: continuous burning pain rated 8/10 on the Numeric Rating Scale (NRS) localized to the L5–S1 dermatome of the right foot, and intermittent sharp, tearing pain in the lower leg, especially at night, requiring regular intramuscular tramadol injections. Physical examination revealed grade 4 right foot muscle strength on manual muscle testing and marked hyperalgesia without erythema or swelling. Despite multimodal pharmacologic therapy, including acetaminophen 650 mg and tramadol 75 mg twice daily, pregabalin 450 mg daily, celecoxib 200 mg twice daily, and topical capsaicin, her symptoms persisted. Ultrasound-guided psoas compartment block and caudal epidural block provided only minimal relief, reducing her NRS pain score from 8 to 7. Electrodiagnostic testing confirmed right sacral plexopathy, consistent with her clinical symptoms.

Given the refractory nature of her neuropathic pain, rTMS was considered as an adjunctive treatment. Because the patient was a minor, particular attention was paid to safety monitoring. The stimulation protocol adhered strictly to established international safety guidelines for rTMS. No adverse effects such as headache, seizure, mood instability, or cognitive changes were observed during or after treatment, supporting the safety and tolerability of HF-rTMS in this adolescent patient. She underwent one session per day for 10 consecutive days. Each session consisted of 30 trains of 10 Hz stimulation (5 s per train, 25 s inter-train intervals), totaling 1500 pulses. A figure-of-eight coil (70 mm) connected to a Magstim Rapid [2] stimulator (Magstim Co., Whitland, UK) was positioned tangentially over the vertex to target the motor cortex representation of the lower extremity. Stimulation intensity was set at 110% of the resting motor threshold, determined as the lowest intensity evoking motor evoked potentials > 50 µV in ≥5 of 10 trials from the flexor pollicis brevis muscle. The patient was comfortably seated in a chair or wheelchair during treatment.

Pain severity and characteristics were assessed using the Short-Form McGill Pain Questionnaire (SF-MPQ) and the Neuropathic Pain Symptom Inventory (NPSI). Depressive symptoms were measured using the Beck Depression Inventory (BDI), and QoL was evaluated with the EQ-5D-5L instrument. Assessments were conducted at baseline, immediately after completion of rTMS, and again at 10-week follow-up (long-term FU). Following rTMS treatment, the patient demonstrated progressive improvement.

## 3. Results

**Figure 1:**
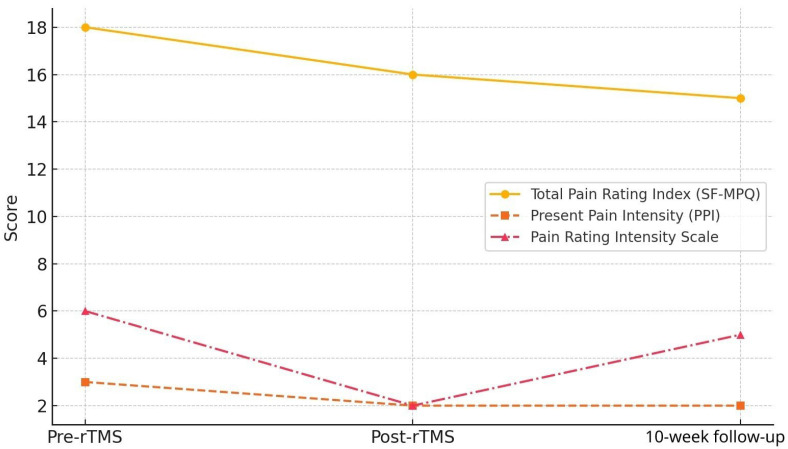
This figure shows the changes in pain intensity before rTMS, immediately after the treatment course, and at the 10-week follow-up. The Short-Form McGill Pain Questionnaire scores and the Numeric Rating Scale score decrease after rTMS, indicating an immediate analgesic effect, with partial maintenance of improvement at 10-week follow-up.

The SF-MPQ total score decreased from 18 at baseline to 16 post-treatment and to 15 at 10-week follow-up. The present pain intensity score improved from 3 to 2 and remained at 2. The NRS pain score improved from 8 before rTMS to 2 immediately after treatment, with a slight increase to 5 at 10-week follow-up (Figure 1). Although there were minor discrepancies between pain scales (e.g., modest change in SF-MPQ total score versus more pronounced NRS reduction), such variability likely reflects differences in scale constructs, with NRS capturing overall pain intensity and SF-MPQ incorporating qualitative pain descriptors.

The NPSI total score decreased from 53 to 52 after treatment to 40 at the 10-week follow-up, with pain quality scores declining from 43 to 44 and then to 39 (Table 1). While the immediate post-treatment reduction in NPSI total score was modest, the marked improvement observed at 10-week follow-up suggests a delayed modulation of neuropathic pain characteristics, which may reflect progressive central neuromodulatory effects.

**Figure 2:**
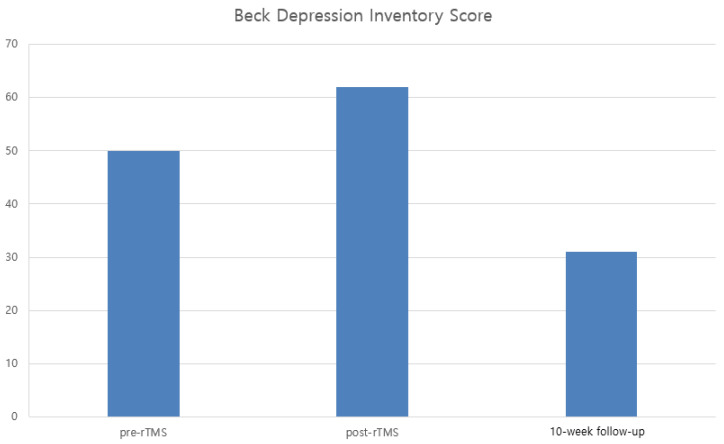
This figure shows the changes in depressive symptoms assessed by the Beck Depression Inventory before rTMS, immediately after treatment, and at the 10-week follow-up. The depressive symptoms initially increase after rTMS but improve markedly at 10-week follow-up.

The BDI score initially worsened from 50 to 62 immediately after rTMS but subsequently improved markedly to 31 at the 10-week follow-up (Figure 2). The transient increase in BDI score immediately after treatment may have been influenced by psychological stress related to hospitalization, fluctuating pain perception, or expectation-related factors rather than a direct adverse effect of rTMS.

These findings suggest a potential secondary improvement in depressive symptoms. Health-related QoL also improved, as the EQ-5D-5L index increased from 0.468 to 0.728 and remained stable at 0.727, while the level sum score decreased from 16 to 11 and stayed at 11 throughout the follow-up period (Table 2). The magnitude of improvement in the EQ-5D-5L index (increase of 0.26) may be considered clinically meaningful, as changes greater than 0.1 are generally regarded as relevant in chronic pain populations. These findings suggest that rTMS provides not only immediate but also persistent long-term improvements in health-related QoL.

## 4. Discussion

Traumatic injury to the LSP is relatively uncommon due to its deep anatomical location within the pelvis. Nevertheless, high-energy trauma such as motor vehicle accidents, gunshot wounds, and complex pelvic or acetabular fractures may lead to LSP involvement [3,4]. Prior studies have shown that the incidence of LSP increases substantially in patients with sacral fractures or sacroiliac joint disruptions, likely due to the anatomical proximity of the plexus to these structures. For instance, a single-center review reported an incidence of 0.7% among all pelvic or acetabular fractures, but this rate rose to 2.03% in cases involving sacral fractures [4]. Similarly, a large retrospective electrodiagnostic study identified 29 cases of traumatic LSP, most frequently associated with motor vehicle accidents and gunshot injuries [3]. Consistent with these findings, the etiology in our case was linked to extensive pelvic trauma involving the acetabulum, sacrum, and pelvic ring, underscoring the vulnerability of the LSP in severe pelvic injuries.

Neuropathic pain following traumatic LSP poses substantial diagnostic and therapeutic challenges. Neuropathic pain is a common and debilitating condition that substantially impairs quality of life (QoL) and often responds inadequately to conventional pharmacological and interventional treatments [6,7]. A structured, stepwise treatment paradigm has been recommended, ranging from first-line agents such as tricyclic antidepressants, gabapentinoids, and serotonin–norepinephrine reuptake inhibitors to more invasive approaches, including epidural injections, sympathetic blocks, adhesiolysis, neuromodulation, and ultimately low-dose opioids as a last resort [9]. However, the limited durability and efficacy of these treatments highlight the need for alternative therapeutic strategies.

Repetitive transcranial magnetic stimulation is a non-invasive neuromodulatory technique that has gained increasing interest for the management of neuropathic pain [10]. HF-rTMS targeting the primary motor cortex is thought to modulate sensory, affective, and descending inhibitory pain pathways. Non-invasive brain stimulation techniques, including rTMS, may provide clinically meaningful analgesic effects by modulating cortical excitability and descending pain inhibitory pathways, supporting their use as adjunctive therapies for refractory neuropathic pain [15]. Proposed mechanisms include elevation of pain thresholds through cortical–thalamic projections, modulation of affective circuits via the insula and anterior cingulate cortex [16,17], and activation of descending pain inhibitory pathways via the periaqueductal gray and rostroventromedial medulla [17]. Neuronavigation-guided stimulation has demonstrated longer-lasting analgesic effects compared with non-guided stimulation [10]. Increases in beta-endorphin levels and the reversal of analgesic effects after naloxone administration further support the involvement of endogenous opioid pathways [18], while preclinical studies suggest influences on NMDA and AMPA/kainate receptors [10]. In summary, high-frequency stimulation of the primary motor cortex may enhance endogenous pain inhibitory circuits and modulate central pain processing pathways rather than acting directly at the site of peripheral nerve injury. Although the existing literature suggests that the analgesic benefit of HF-rTMS generally persists for up to one week post-treatment [19], our case demonstrated sustained pain reduction for at least 10 weeks, suggesting a potentially longer therapeutic window in traumatic LSP.

In our patient, rTMS was associated with a marked immediate reduction in the NRS pain score (6 to 2), partial maintenance of analgesic benefit at 10 weeks, substantial long-term reduction in NPSI score (53 to 40), and clinically meaningful improvement in QoL as reflected by the EQ-5D-5L index. Although depressive symptoms transiently worsened immediately after treatment, the long-term reduction in BDI score (62 to 31) suggests a secondary mood benefit potentially mediated by pain reduction.

The safety profile of rTMS is well established. Reported adverse effects are typically mild and transient, including headache, scalp discomfort, and fatigue, while serious events such as seizures are exceedingly rare when safety guidelines are followed [11]. In the present case, the patient tolerated the entire treatment course without any adverse events, reinforcing the clinical safety and feasibility of HF-rTMS in individuals with traumatic neuropathic pain. The safety profile of rTMS in pediatric and adolescent populations has been increasingly supported by recent clinical studies, which report low rates of serious adverse events when standard safety protocols are followed. Our case further supports the tolerability of HF-rTMS in a minor patient with traumatic neuropathic pain [20].

Given the strong bidirectional relationship between chronic pain and mood disturbances, improvements in depressive symptoms following rTMS are clinically meaningful. Chronic pain and depression share overlapping neuronal circuits within the prefrontal cortex, insula, limbic system, and anterior cingulate cortex [21,22,23]. Although our stimulation protocol did not target traditional sites for the treatment of depression, the observed improvement in BDI scores may reflect secondary benefits from pain reduction rather than direct antidepressant effects. In addition, QoL improvements, as measured by the EQ-5D-5L, align with several previous studies reporting functional gains following rTMS [24], though conflicting evidence remains. Variability across studies likely reflects differences in pain etiologies, stimulation protocols, and outcome measures.

This study has several limitations. As a single-case report, the absence of a control group, randomization, and blinding limits causal inference. The placebo effect cannot be excluded. Psychological variability may influence patient-reported mood and QoL measures despite the use of validated instruments. Furthermore, although our stimulation protocol was based on existing evidence from chronic neuropathic pain studies, it was not specifically optimized for LSP. Future research should focus on tailoring neuromodulation parameters for LSP-related neuropathic pain and conducting larger controlled studies to verify the long-term efficacy of HF-rTMS. The limitation of this study relates to stimulation target localization. rTMS was delivered using a standard scalp landmark-based approach rather than neuronavigation. Although neuronavigation may improve targeting precision, landmark-based localization remains commonly used in clinical practice and provides acceptable reliability. Nevertheless, the absence of neuronavigation may introduce variability in stimulation targeting and should be considered when interpreting the observed therapeutic effects.

## 5. Conclusions

In conclusion, repetitive transcranial magnetic stimulation may offer a safe and effective therapeutic option for managing neuropathic pain following traumatic LSP. Our findings suggest that HF-rTMS not only alleviates neuropathic pain but may also contribute to improvements in depressive symptoms and overall QoL, underscoring its potential as a valuable adjunctive therapy when conventional treatments provide insufficient relief. Particularly in young patients with limited treatment options and concerns regarding long-term pharmacotherapy, HF-rTMS may represent a clinically meaningful adjunctive intervention with a favorable safety profile.

## Figures and Tables

**Table 1:** Neuropathic Pain Symptom Inventory.

Parameters	Pre-rTMS	Post-rTMS	10-Week Follow-Up
total score	53	52	40
time score	10	8	1
pain quality score	43	44	39
superficial pain	5	0	3
deep pain	1.5	0	2
paroxysmal pain	6	6	5
evoked pain	5	7	4
dysesthesia	4	5.5	5

**Table 2:** EQ-5D-5L Scores.

	Pre-rTMS	Post-rTMS	10-Week Follow-Up
EQ 5D-5L index	0.468	0.728	0.727
Level sum score	16	11	11
Level frequency score	11,102	12,200	12,200

## Data Availability

The original contributions presented in this study are included in the article. Further inquiries can be directed to the corresponding author.

## Abbreviations

LSP, lumbosacral plexopathy; QoL, quality of life; rTMS, repetitive transcranial magnetic stimulation; MRI, magnetic resonance imaging; IASP, International Association for the Study of Pain; TMS, transcranial magnetic stimulation; HF-rTMS, high-frequency repetitive transcranial magnetic stimulation; NRS, numeric rating scale; SF-MPQ, Short-Form McGill Pain Questionnaire; NPSI, Neuropathic Pain Symptom Inventory; BDI, Beck Depression Inventory; EQ-5D-5L, EuroQol five-dimension five-level questionnaire; MEP, motor evoked potential; NMDA, N-methyl-D-aspartate; AMPA, α-amino-3-hydroxy-5-methyl-4-isoxazolepropionic acid.
